# Low-Noise Dynamic Reconstruction for X-Ray Tomographic Perfusion Studies Using Low Sampling Rates

**DOI:** 10.1155/2009/108028

**Published:** 2010-01-17

**Authors:** Pau Montes, Günter Lauritsch

**Affiliations:** ^1^Interdisciplinary Center for Scientific Computing (IWR), University of Heidelberg, Im Neuenheimer Feld 368, 69120 Heidelberg, Germany; ^2^Siemens AG Healthcare Sector, P.O. Box 1266, 91294 Forchheim, Germany

## Abstract

Functional imaging based on tomographic X-ray imaging relies on the reconstruction of a temporal sequence of images which accurately reproduces the time attenuation curves of the tissue. The main constraints of these techniques are temporal resolution and dose. Using current techniques the data acquisition has to be performed fast so that the dynamic attenuation values can be regarded as static during the scan. Due to the relatively high number of repeated scans the dose per single scan has to be low yielding a poor signal-to-noise ratio (SNR) in the reconstructed images. In a previous publication a temporal interpolation scheme in the projection data space was relaxing the temporal resolution constraint. The aim of this contribution is the improvement of the SNR. A temporal smoothing term is introduced in the temporal interpolation scheme such that only the physiologic relevant bandwidth is considered. A significant increase of the SNR is achieved. The obtained level of noise only depends on the total dose applied and is independent of the number of scans and the SNR of a single reconstructed image. The approach might be the first step towards using slowly rotating CT systems for perfusion imaging like C-arm or small animal CT scanners.

## 1. Introduction

The application of tomographic X-ray techniques to carry out perfusion studies relies on the reconstruction of a sequence of images which accurately reproduces the dynamics of the flow of an injected contrast agent. The temporal evolution of the attenuation coefficient corresponding to a voxel is denoted as *time attenuation curve* (TAC) or *perfusion signal* and is proportional to the concentration of contrast agent in the region occupied by said voxel. Hence, the temporal evolution of the gray value of a voxel is proportional to the temporal evolution of the average concentration of contrast agent within the voxel. TACs are used as input for algorithms based on a tracer kinetic model which compute functional parameter maps. Several approaches have been presented in the literature for this purpose. We refer the reader to [1] for an overview of these methods. The obtained maps provide the physician with physiological information related to blood supply to the tissues in the ROI which is crucial for the diagnosis. 

 One of the main constraints posed by these techniques is the temporal resolution; that is, the data acquisition-reconstruction process has to be capable of reproducing the perfusion signals. For this reason, up to now, data acquisition is performed fast enough to assume that the attenuation values are constant during data acquisition. Another important issue are dose considerations. Due to the relatively high number of repeated scans in a perfusion study the dose per single scan has to be low yielding a poor signal to noise ratio in the reconstructed images. The noise level can even be higher than the level of contrast enhancement caused by contrast agent flow. Under such conditions, the detection of perfusion signals becomes a challenging issue. This aspect will become even more critical with the introduction of large area detectors where a larger area of the patient is exposed. 

 Currently, only CT scanners allow a sampling rate high enough to assume constant attenuation during acquisition and therefore the only clinical application of tomographic X-ray techniques to perform perfusion studies is perfusion Computed Tomography (perfusion CT). This technique has already found its way into clinical routine where it represents, together with MRI, one of the primary imaging techniques for the diagnosis of patients with symptoms of stroke. It presents several advantages over other techniques to perform perfusion studies as MRI which include the widespread availability of CT scanners, an easier access to the patient and lower cost [2]; moreover, several studies indicate that the potential of this technique is not yet fully exploited [3–5]. In this paper we propose an acquisition-reconstruction scheme which tackles both the temporal resolution and the noise problem making it suitable for perfusion studies with other tomographic X-ray techniques. 

 In [6] we introduced an algorithm with increased temporal resolution under the assumption of no motion, which weakened the requirement of fast data acquisition. This algorithm (TIA-TFDK: Temporal Interpolation Approach Tent Feldkamp Davis Kress) is based on a convolution and backprojection approach for circular trajectories and reconstructs an intermediate volume image denoted as partial block backprojection (PBB) from only that part of the projection data that are acquired in a time period of a given length. The final volume image at a certain time is computed by accumulating a complete set of PBBs of the same time, thus reducing data inconsistencies. For this purpose a temporal spline interpolation approach is used to estimate the value of a PBB at any desired time. Image acquisition has to be fast enough such that the PBBs satisfy Nyquist's sampling condition. However, the TIA-TFDK algorithm provides a fixed temporal resolution for a given rotation time of the scanner and it does not take noise into account. In order to tackle this two aspects, we introduce in this a simple modification of the TIA-TFDK algorithm which exploits the fact that perfusion signals are essentially low pass. An estimation of the maximum frequency of the fastest perfusion signal in the region of interest is used as a priori information. By substituting the interpolation step for a more flexible estimation scheme which allows to choose the temporal bandwidth of the output sequence, higher frequency components containing mainly noise are suppressed and thus the SNR is improved. In our modification, which we call Temporal Smoothing Approach-TFDK (TSA-TFDK), we propose to use smoothing splines as a natural evolution from the interpolation splines scheme used in [6]. With the proposed algorithm the temporal bandwidth is an additional parameter which is independent of the rotation time. We also justify theoretically that under certain assumption (no motion and only quantum noise) the image quality can be shown to be the same independently of the rotation time. 

 This algorithm opens the possibility to use other tomographic X-ray techniques for perfusion studies as, for example, small animal CT scanners and represents a first step toward the application of C-arm systems for perfusion studies. However, the assumption of no motion limits the possible clinical applications to fixed patients. 

 We start with the setting of our dynamic reconstruction problem. We then describe the low-noise estimation of time dependent projections in the presence of noise when the only a priori information available is the signal's bandwidth. We describe a practical implementation of this low-noise estimation with polynomial spline filters. Subsequently, we apply these ideas to the dynamic reconstruction problem and describe the TSA-TFDK approach for dynamic reconstruction. Finally, we present a numerical simulation and provide an illustrative example of the effect of the noise reduction on clinical data.

## 2. Problem Setting

**Figure 1:**
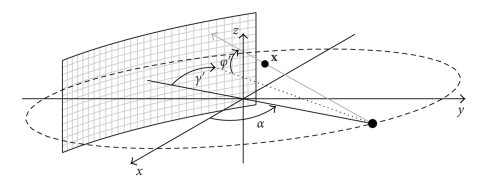
Scheme of a CT scanner in cone-beam geometry with a cylindrical detector.

A source-detector arrangement with a cylindrical detector as shown in Figure 1 rotates around the *z* axis with a constant angular speed *ω* on a circular trajectory. The angular speed *ω* determines the rotation time *T*_2*π*_, *T*_2*π*_ = 2*π*/*ω*. The plane containing the source trajectory is denoted as *x* *y* plane. We assume that the source is situated at the angular position *α* = 0 at *t* = 0; hence, the cone-beam projection *P*_*α*_(*γ*, *φ*, *t*_*α*_) is acquired at *t*_*α*_ = *α*/*ω*. *γ* and *φ* denote the fan and cone-angles, respectively, and *γ*′(*α*, **x**), and *φ*′(*α*, **x**), the fan and cone-angles which determine the ray passing through **x**, where **x** ∈ ℝ^3^ denotes the spatial coordinate, for a given angular position *α*. The object is represented by a time dependent spatial distribution of an attenuation coefficient *μ*(**x**, *t*), where *t* denotes time, and is located within a cylinder of radius ||**x**_max_||, that is,

(1) $$\begin{array}{c} \mu \left(x,t\right)=0\;\text{for}||x||\geq ||x_{\max\;}||. \end{array}$$

We concentrate in changes of the attenuation coefficient caused by the change in the composition of a substance as in the case of contrast agent flow. We assume that neither motion nor deformation occurs. 

 The source rotates continuously during a total acquisition time of *T*_tot_ and during this time a total dose *D*_tot_ is applied. We assume that the total dose is distributed uniformly among all projections acquired during the acquisition time. We use as an estimation of the dose applied the mAs product, which is a reasonable measure to compare reconstruction algorithms in terms of dose efficiency as long as the input data are obtained with the same scanner [7]. During its continuous rotation the source may be switched off during regular periods of time. 

 We also assume that the only source of noise is quantum noise. This is a reasonable assumption for objects with small sections, for example, in neurological applications. 

 Our goal is the estimation of *μ*(**x**, *t*) at temporal increments of *T*_fr_ during a total time of *T*_tot_.

## 3. Low-Noise Estimation in Dynamic X-Ray Tomographic Imaging

### 3.1. Low-Noise Estimation of Time Dependent Projections

Let us now consider the problem of the estimation of the continuous projection value *p*(*t*) from the noisy measurements *y*[*n*] taken every *T*_*s*_. 

 The continuous projection value *p*(*t*) is an accumulation of time attenuation curves along the direction of the x-ray beam. In order to find an appropriate method to estimate *p*(*t*) it is convenient first to characterize TACs in order to be able to extract the maximum information from the measurements. The model, however, should be as general as possible in order for it to be valid both in pathologic and nonpathologic cases. The morphology of perfusion signals depends on the patient, on the tissue, and on the injection parameters (amount of contrast agent, injection rate, etc.) so that it is very variable. The task to find a model that represents their variety is a very challenging one. The model we propose is motivated by the linear systems approach for the modeling of tracer kinetics [8]. According to this approach, the time-attenuation curve in a tissue is obtained by convolution of the time-attenuation curve of the input artery with the impulse response of the tissue which is essentially a low-pass filter. The flow through several tissues is modeled by successive convolutions. Additionally, prior to reaching tissue, venously injected contrast agent goes through the cardio-pulmonary system which has a very strong low-pass filtering effect. As a consequence of this, TACs are very smooth curves. In Fourier domain, this translates into a Fourier transform that can be neglected over a certain threshold which we denote as *ν*_max_. Moreover, the injection profile is usually a rectangular pulse; thus, the power spectrum of TACs is concentrated around 0 Hz. As an example, typical values of *ν*_max_ measured in the brain at the *arteria cerebri anterior* with an injection rate of 20 mL/s and an injected volume of 50 mL are ≤0.15 Hz [9]. 

**Figure 2:**
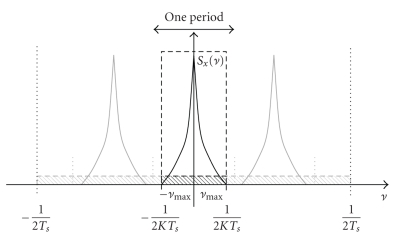
Principle of low-pass filtering to obtain a low-noise sequence. The ideal low-pass filter adapted to the signal covers the frequency band ] − *ν*_max_, *ν*_max_[ (black dashed). The shaded area indicates the noise power density. Light shaded indicates masked out noise; whereas dark shaded indicates the noise in the frequency band of the signal. The light shaded spectra are repetitions of the spectrum of the signal due to sampling.

 According to the model presented in Appendix A, the power spectral density of quantum noise is constant over all frequencies. The natural conclusion is then that suppressing frequency components over |*ν*_max_|, the signal is preserved and only noise is eliminated, increasing thus the signal to noise ratio. The maximum enhancement of the SNR is obtained by using a continuous ideal low-pass filter with cut-off frequency *ν*_max_ for the estimation. This principle works only if the sampling condition is fulfilled

(2) $$\begin{array}{c} \nu_{\max\;}<\frac{1}{2T_{s}}, \end{array}$$

which ensures that the repetitions of the frequency spectrum (light shaded spectra in Figure 2) do not overlap. 

The variance of the noise after the estimation *σ*_Est_^2^ is obtained by integrating the power spectral density of the noise in the frequency band ] − *ν*_max_, *ν*_max_[. Since the power spectral density of noise is constant equal to $\bar{\varepsilon^{2}}T_{s}$ and the frequency response of the ideal low-pass filter is constant in ] − *ν*_max_, *ν*_max_[, we get

(3) $$\begin{array}{c} \sigma_{\text{Est}}^{2}=2\nu_{\max\;}\bar{\varepsilon^{2}}T_{s}. \end{array}$$

Note that in order to come to (3) we have only used the hypotheses of quantum white noise (see Appendix A), bandlimited signal, and constant total dose. In the following we consider the influence of dose settings in the noise variance. 

 According to the definition of dose, the mAs product dose is linearly accumulated. Hence, the total dose applied *D*_tot_ is

(4) $$\begin{array}{c} D_{\text{tot}}=D_{p}N_{\text{rot}}N_{\alpha }, \end{array}$$

where *D*_*p*_ is the dose applied to measure each projection, *N*_rot_ is the number of rotations with activated X-ray source, and *N*_*α*_ is the number of projections acquired per rotation. Every projection is measured once during a rotation, hence *N*_rot_, *T*_*s*_, and the total acquisition time are related by

(5) $$\begin{array}{c} N_{\text{rot}}=\frac{T_{\text{tot}}}{T_{s}}. \end{array}$$

Note that *T*_*s*_ need not to be *T*_2*π*_ but might be a multiple of it. Since the total dose is distributed uniformly among all projections acquired, the dose applied to measure a projection is

(6) $$\begin{array}{c} D_{p}=\frac{D_{\text{tot}}}{N_{\text{rot}}N_{\alpha }}. \end{array}$$

It is a well-known fact that for quantum noise the mean square value is inversely proportional to the dose applied $\bar{\varepsilon^{2}}\propto 1/D_{p}$. Using (5) and (6), we get

(7) $$\begin{array}{c} \bar{\varepsilon^{2}}\propto \frac{T_{\text{tot}}N_{\alpha }}{T_{s}D_{\text{tot}}}. \end{array}$$

Finally, using (3) we get

(8) $$\begin{array}{c} \sigma_{\text{Est}}^{2}\propto \frac{\nu_{\max\;}}{D_{\text{tot}}}T_{\text{tot}}N_{\alpha }. \end{array}$$

This expression can be interpreted as follows. The estimation with a continuous low-pass filter exploits the redundancy in the signal to reduce noise, and 1/*ν*_max_ is a good indicator for signal redundancy. Hence, for a given total dose *D*_tot_ distributed during a total acquisition time *T*_tot_ and acquiring *N*_*α*_ projections per rotation, the higher the redundancy is (i.e., the lower *ν*_max_), the stronger the achieved noise reduction will be. Note that *σ*_Est_^2^ does not depend on *T*_*s*_. Thus the same level of noise will be obtained by acquiring a few scans with a high dose per view or a higher amount of scans with lower dose per view, as long as condition (2) is fulfilled.

### 3.2. Low-Noise Estimation with Polynomial Spline Filters

As stated in the introduction, we use the TIA-TFDK algorithm from [6] as a starting point for our new approach. Hence, the most natural choice to approximate the ideal low-pass filters described in the previous section is to use polynomial spline filters, since this will imply a minor modification in the algorithm. 

 An ideal low-pass filter has an infinite support in temporal domain and decays very slowly (∝ 1/|*t*|) so that samples that lie far from the value to be estimated will still contribute significantly to it. This is very inefficient for practical purposes. Polynomial spline filters are appropiate to approximate ideal low-pass filters for our purposes for two main reasons. First, from [6] as a starting point for our new approach, hence this is a natural choice since it implies a minor modification in the algorithm. Additionally, polynomial splines provide a viable compromise between accuracy and efficiency. The filter kernels are quite local and converge rapidly to the ideal low-pass kernel when the order *n* of the polynomial splines increases. Given a set of samples, by properly choosing the coefficients of the polynomial spline basis functions (B-splines), estimations of any degree of smoothing and, in the extreme, of no smoothing at all (interpolation) can be performed [10, 11]. In this section we describe how temporal smoothing with polynomial splines can be interpreted as a low-pass filtering in time. 

 We denote with *y*[*k*] the temporal noisy samples of a given signal *p*(*t*) taken every *T*_*s*_. Throughout this section *t*′ denotes time normalized by the sampling interval *T*_*s*_, that is, *t*′ = *t*/*T*_*s*_; the corresponding dimensionless frequency is denoted by *ν*′ = *ν* *T*_*s*_. We approximate the continuous signal *p*(*t*′) by a polynomial spline function of order *n*: *s*_*λ*_^*n*^(*t*′), where *λ* indicates the degree of smoothing. According to Schönberg's theorem (see [10]), *s*_*λ*_^*n*^(*t*′) can be expressed as

(9) $$\begin{array}{c} s_{\lambda }^{n}\left(t^{\prime }\right)=\underset{k\in \mathbb{Z}}{\sum }c_{\lambda }\left[k\right]\beta^{n}\left(t^{\prime }-k\right), \end{array}$$

where *β* ^*n*^(*t*′) are the polynomial B-splines and *c*_*λ*_[*k*] the B-spline coefficients. The polynomial B-splines are uniquely determined by its order *n* so that, for a given order *n*, we only need to find the coefficients *c*_*λ*_[*k*]. For this purpose we minimize the following functional:

(10) $$\begin{array}{c} F\left\{c_{\lambda },y\right\}=\underset{k\in \mathbb{Z}}{\sum }{\left(y[k]-s_{\lambda }^{n}[k]\right)}^{2}+\lambda {||D^{L}s_{\lambda }^{n}(t)||}_{L_{2}}^{2}, \end{array}$$

where *n* = 2*L* − 1, *D* ^*L*^ denotes the *L*th derivative and ||·||_*L*_2__ the *L*_2_ norm. The first term in (10) forces the estimated function to be close to the sampled values at the sampling points. The second term is a regularity constraint which favors a smooth estimation of the signal. It is controlled by the smoothing parameter *λ*. For *λ* = 0, *F*{**c**_***λ***_, **y**} = 0 if *s*_*λ*_^*n*^[*k*] = *y*[*k*], which is the interpolation condition. For larger values of the smoothing parameter, the smoothness constraint might determine a curve not passing through the sampled values. If we denote *b* ^*n*^[*k*] = *β* ^*n*^(*k*), it can be shown (see [11]) that the coefficients of the *n*th-order polynomial spline that minimize (10) can be computed as

(11) $$\begin{array}{c} {\hat{C}}_{\lambda }\left(e^{i2\pi \nu^{\prime }}\right)=\frac{\hat{Y}\left(e^{i2\pi \nu^{\prime }}\right)}{{\hat{B}}^{n}\left(e^{i2\pi \nu^{\prime }}\right)+\lambda {\left(-e^{i2\pi \nu^{\prime }}+2-e^{-i2\pi \nu^{\prime }}\right)}^{L}}, \end{array}$$

where ${\hat{C}}_{\lambda }\left(e^{i2\pi \nu^{\prime }}\right),\hat{Y}\left(e^{i2\pi \nu^{\prime }}\right)$- and ${\hat{B}}^{n}\left(e^{i2\pi \nu^{\prime }}\right)$ denote the Fourier Transform of the discrete sequences *c*_*λ*_[*k*], *y*[*k*], and *b* ^*n*^[*k*], respectively. Substituting the spline coefficients ${\hat{C}}_{\lambda }\left(e^{i2\pi \nu^{\prime }}\right)$ of (11) in the Fourier Transform of (9) we obtain

(12) $${\hat{s}}_{\lambda }^{n}\left(\nu^{\prime }\right)=\hat{Y}\left(e^{i2\pi \nu^{\prime }}\right)\underset{{\hat{\eta }}_{\lambda }^{n}\left(\nu^{\prime }\right)}{\underset{︸}{\frac{{\left(\text{sinc}(\nu^{\prime })\right)}^{n+1}}{{\hat{B}}^{n}\left(e^{i2\pi \nu^{\prime }}\right)+\lambda {\left(-e^{i2\pi \nu^{\prime }}+2-e^{-i2\pi \nu^{\prime }}\right)}^{L}}}},$$

where we have used that ${{\hat{\beta }}^{n}(\nu^{\prime })=(\text{sinc}(\nu^{\prime }))}^{n+1}$. 

 Equation (12) shows that the smoothing operation can be interpreted as the discrete convolution of the samples *y*[*k*] with a continuous polynomial spline low-pass filter *η*_*λ*_^*n*^(*t*′). When *λ* = 0, *η*_0_^*n*^(*t*′) is the polynomial spline interpolator of order *n* and it verifies lim _*n*→*∞*_ *η*_0_^*n*^(*t*′) = sinc(*t*′) [12]. 

**Figure 3:**
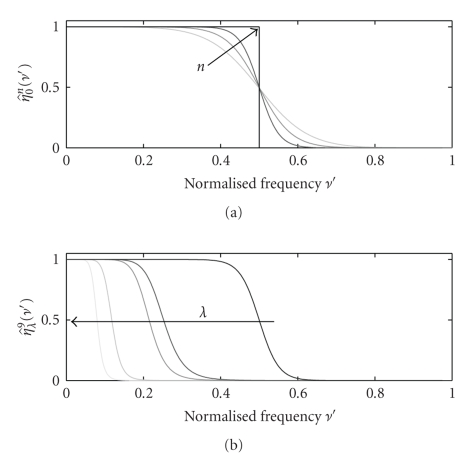
Frequency response of the polynomial spline filter *η*_*λ*_^*n*^(*t*′). (a) *λ* = 0 and orders *n* = 3, 5 and 9 (black arrow indicates increasing order). (b) *n* = 9 and *λ* = 0,0.01,0.05,20 and 1000 (black arrow indicates increasing value of *λ*). Frequency *ν*′ is normalized by the sampling frequency.

 We denote as cut-off frequency the frequency *ν*_*c*_′ at which the frequency response of the filter falls to half of the maximum, that is, ${{\hat{\eta }}_{\lambda }(\nu '}_{c})=0.5$. Figure 3 shows the frequency responses of different polynomial spline filters *η*_*λ*_^*n*^(*t*′) for different values of *n* and *λ*. By properly choosing these parameters we can obtain good approximations to the ideal low-pass filters used in Appendix A. In practice, *n* is responsible for the sharpness of the edges of the frequency response, that is, how close the filter is to the ideal low-pass filter (Figure 3(a)) and *λ* for the position of the cut-off frequency (Figure 3(b)). 

**Figure 4:**
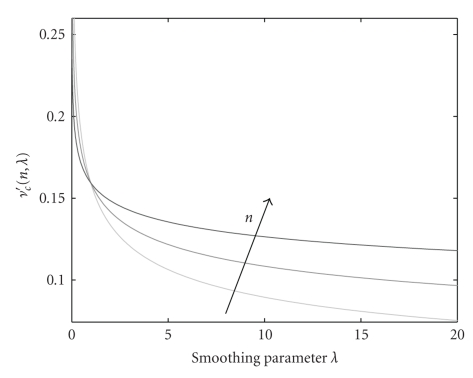
Dependency of the cut-off frequency of polynomial spline filters with the smoothing parameter *λ* for *n* = 3, 5, and 9 (black arrow indicates increasing order).

Even if *λ* is responsible for the position of the cut-off frequency, this dependency is different for different orders *n*. This is illustrated in Figure 4. For *λ* = 0, *ν*_*c*_′ = 0.5 for all *n* values. The cut-off frequency decreases slower for lower orders until *λ* ≈ 1. After this value, lower orders decrease much faster. For high values of *λ* the decay is very slow and therefore large increases in the smoothing parameter provide only very small reduction of the cut-off frequency. The value of *λ*, the normalized cut-off frequency *ν*_*c*_′ and the spline of order *n* are related by [13]

(13) $$\lambda ={\left(2\pi \nu_{c}^{\prime }\right)}^{-n-1}-\pi^{-n-1}.$$

Contrary to the frequency response of the ideal low-pass filter, the frequency response of polynomial spline filters starts decaying already for frequencies under *ν*_*c*_′. The proportion of the frequency interval [0, *ν*_*c*_′] which can be reproduced accurately depends mainly on the order of the splines *n*. For the purpose of this paper we will use *n* = 9 as an approximation for the ideal low-pass filter. Figure 3 shows that for *n* = 9 the frequency response is almost constant until approximately 0.8*ν*_*c*_′. As a consequence, we will use hereafter the following expression to calculate the cut-off frequency to ensure that the frequency range from 0 to *ν*_max_ remains unaltered after filtering

(14) $$\nu_{c}=\frac{\nu_{\max\;}}{0.8}\to \nu_{c}^{\prime }=\frac{\nu_{\max\;}T_{s}}{0.8}.$$

In Section 3.1, we provided an expression for the estimation of the variance of the filtered sequence. We will derive now an expression for the case where polynomial spline filtering is used instead of ideal filtering. The power spectrum of a filtered stationary process is obtained by integrating the product of the power spectrum of the process with the square of the absolute value of the frequency response of the filter [14]. Since we used an ideal low-pass filter, the integral from −*ν*_max_ to *ν*_max_ could simply be substituted by 2*ν*_max_ times the value of the power spectral density. If we use a spline low-pass filter this is no longer true. We now have for (3)

(15) $$\sigma_{\text{Spl}}^{2}=\bar{\varepsilon^{2}}T_{s}\int_{-\infty }^{+\infty }{\left|{\hat{\eta }}_{\lambda }^{n}(\nu^{\prime })\right|}^{2}d\nu^{\prime }.$$

**Figure 5:**
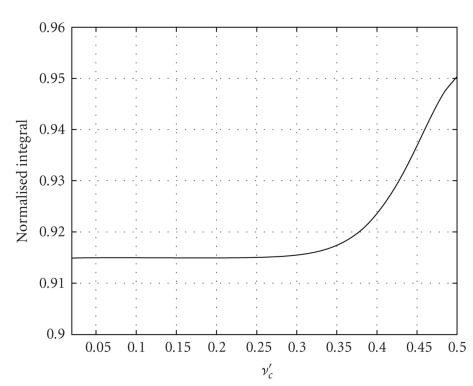
Value of $\int |{\hat{\eta }}_{\lambda }^{9}\left(\nu^{\prime }\right){|}^{2}d\nu^{\prime }$ normalized to 2*ν*_*c*_′.

The value of the integral depends on *n* and *λ*. In Figure 3, we show the frequency response of *η*_*λ*_^9^(*t*′) for different values of *λ*. Qualitatively it is clear that all these filters are very close to an ideal low-pass filter with the corresponding cut-off frequency. Hence, the value of the integral must be close to 2*ν*_*c*_′. In Figure 5 we show the ratio

(16) $$\frac{\int_{-\infty }^{+\infty }{\left|{\hat{\eta }}_{\lambda }^{9}(\nu^{\prime })\right|}^{2}d\nu^{\prime }}{2\nu_{c}^{\prime }}$$

for values of *ν*_*c*_′ ∈ [0.02,0.5]. The values are all between 0.914 and 0.95. We would like to have a simple rule of thumb to estimate the value of the variance of a sequence filtered with a low-pass spline filter. For this purpose, we propose to use the average value of the curve in Figure 5, 0.92 as a representative value and then add the factor 0.92/0.8 to (3) as a correction factor for filtering with splines. This yields

(17) $$\begin{array}{c} \sigma_{\text{Spl}}^{2}\approx 2.3\bar{\varepsilon^{2}}T_{s}\nu_{\max\;}. \end{array}$$

## 4. Temporal Smoothing Approach-TFDK

In this section we discuss the modification of the TIA-TFDK algorithm to exploit the ideas presented in the previous sections. For the sake of simplicity we initially assume that the source is always switched on during acquisition time, that is, it operates in continuous acquisition mode. 

**Figure 6:**
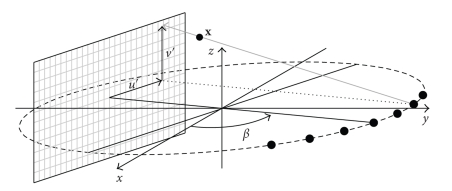
Scheme of the the rebinned fan-parallel beam geometry.

 The TIA-TFDK algorithm can be seen as a dynamic extension of the T-FDK algorithm for cone-beam reconstruction on circular trajectories described in [15]. We first introduce this algorithm to which we have added the temporal dependence of the object. Unclear relationships between time variables will be explained further on. The T-FDK algorithm consists in first rebinning the cone-beam projections to a fan-parallel beam (see Figure 6)

(18) $$\begin{array}{c} P_{\alpha }\left(\gamma ,\phi ,t_{\alpha }\right)\to P_{\beta }^{b}\left(u,v,t_{\beta }\right), \end{array}$$

and then performing filtering and backprojection on the rebinned projections:

(19) $$\begin{array}{cc} Q_{\beta }\left(u,v,t_{\beta }\right) & =\int_{-u_{\max\;}}^{u_{\max\;}}\left(P_{\beta }^{b}\left(s,v,t_{\beta }\right)w\left(s,v\right)\right)g\left(u-s\right)ds,\\ \mu^{r}\left(x,t_{\pi }\right) & =\frac{1}{2}\int_{0}^{2\pi }Q_{\beta }\left(u^{\prime }\left(x,\beta \right),v^{\prime }\left(x,\beta \right),t_{\beta }\right)d\beta , \end{array}$$

where *w*(*u*, *v*) is a weighting function which only depends on the detector coordinates and (*u*′(**x**, *β*), *v*′(**x**, *β*)) are the coordinates of the detector pixel where the ray passing through **x** intersects the detector for the projection at projection angle *β* (see Figure 6). 

 Both the rebinning and the backprojection steps include approximations since in both, projections acquired at different times are used to compute a value which is associated to a unique time. During the rebinning step, in order to calculate the rebinned value at projection angle *β*, cone-beam projections acquired during the time interval *t*_*α*_ ∈ [*t*_*β*_ − *γ*_max_, *t*_*β*_ + *γ*_max_] are used, where *γ*_max_ is the maximum fan-angle. Hence, the time uncertainty introduced in the rebinning step depends on the maximum fan-angle, that is, on the diameter of the object, whereas the time uncertainty due to the backprojection step depends on the length of the backprojection interval. We can therefore reduce the uncertainty due to backprojection by dividing the angular interval [0,2*π*] in *N* subintervals of length 2*π*/*N*. We denote the backprojection over these subintervals as partial block backprojections (PBBs) as in [16]. Since every PBB occupies the same amount of memory as the reconstructed volume, taking a lot of PBB intervals has a direct impact on the amount of memory required for the reconstruction. On the other hand, as shown in [6], the uncertainty due to rebinning does not depend on the number of PBBs per rotation *N*; there is a certain threshold *N*_max_, which only depends on the maximum fan-angle, over which the error due to rebinning predominates and makes it not worth increasing *N*, that is, to reduce the length of the backprojection interval. For perfusion signals, [6] shows that with the typical fields of view of most current CT scanners and *N* ≥ *N*_max_, the errors due to the uncertainties introduced by rebinning and backprojection can be neglected when compared to the errors due to resolution or noise. In the TSA-TFDK, the reconstruction of a PBB over several rotations can be interpreted as the sampling of time dependent PBB with a sampling interval *T*_*s*_ = *T*_2*π*_. Furthermore, in the rebinned geometry, for small cone-angles, equivalent rays are acquired every *T*_*s*_ = *T*_*π*_. That is to say, reconstructing PBBs over several rotations provides a series of noisy samples acquired every half-rotation. 

 In Appendix A we assumed that the variances of the projection values do not depend on time and derived a model for the temporal behavior of noise in projections based on this assumption. It should be noted, however, that if this assumption holds for projection values, it automatically holds for filtered projection values and also for linear combinations thereof, for example, to PBBs. Similarly, the statistical independence of the fluctuations at different times is guaranteed for filtered projections and linear combinations of projections as long as the projection sets are not overlapping. As a consequence, the proposed model for the temporal behavior of noise in projections, as a stationary process in time with statistically independent time samples can be also applied to filtered projections and partial block backprojections. Therefore, a low-noise estimation is obtained from the series of noisy samples if these are filtered along the time axis with a continuous polynomial spline filter with cut-off frequency *ν*_*c*_ = *ν*_max_/0.8. According to the sampling rate of 1/*T*_*π*_ obtained when combining the time-series of the *j*th PBB with the time series of the *j* + *N*/2th PBB (computed with equivalent rays), accurate reconstruction is possible as long as

(20) $$\begin{array}{c} \nu_{c}=\frac{\nu_{\max\;}}{0.8}\leq \frac{1}{2T_{\pi }}\to T_{2\pi }\leq \frac{0.8}{\nu_{\max\;}}. \end{array}$$

By filtering the PBBs this way, we can estimate their values at any time and thus reconstruct an image frame at any desired reconstruction time by accumulating *N*/2 PBBs. Contrary to the TIA-TFDK algorithm, the temporal estimation is carried out using polynomial spline filters with a normalized cut-off frequency *ν*_*c*_′ ≤ 0.5, that is, the PBB samples are smoothed along the time axis. 

 Note that (20) is based on the assumption that we use polynomial splines of order *n* = 9. If other values of *n* are used, (20) should be adapted accordingly (see Section 3.2). 

 A detailed description of the steps of the algorithm is provided in Appendix B. 

 Since typically *ν*_max_ < 0.15 Hz (see [9]), the condition (14) translates into the rotation time having to fulfill *T*_2*π*_ < 5.33 s, which is achieved by a wide variety of X-ray tomographic imaging systems. For the choice of *N* the most challenging case according to (20) is when *T*_2*π*_ *ν*_max_ = 0.8, for which we provided the following empirical formula in [6]:

(21) $$\begin{array}{c} N_{\max\;}\approx \frac{\pi }{\sqrt{6}}\frac{1}{\sqrt{0.02+r^{2}/4\Gamma^{2}}}, \end{array}$$

where *r* is the radius of the object and Γ is the radius of the circular trajectory of the source. This *N*_max_ gives the value over which the uncertainty introduced by rebinning predominates over the uncertainty produced by backprojection. 

 A especially interesting case results from the application of the algorithm to scanners with high rotational speeds (e.g., 2 rotations per second) because then using the lowest rotation time of the scanner *T*_2*π*_^min ^, *N*, can be reduced to 1 without significant loss of accuracy. Thus, the temporal estimation is carried out as a postprocessing of the reconstructed sequence.

## 5. Numerical Example

In this section we present an example of low-noise acquisition and reconstruction with the TSA-TFDK algorithm and compare it to a perfusion protocol which is representative of current clinical routine perfusion CT. During this section, we will refer to this protocol as the reference protocol. For the acquisition, 800 projections per rotation were simulated with a cylindrical scanner of 256 channels. A spherical phantom containing 6 spherical inserts with time-dependent attenuation was used. In this phantom, the inserts follow the temporal law

(22) $$\begin{array}{c} \mu_{i}\left(t\right)=C_{i}{\left(t-p_{1}\right)}^{p_{2}}e^{-\left(t-p_{1}\right)/p_{3}}, \end{array}$$

with *p*_1_ = 5 s, *p*_2_ = 2.3, and *p*_3_ = 3 s. This curve fulfills *ν*_max_ ≤ 0.15 Hz and is representative of the temporal evolution of the concentration of contrast agent in vessels and tissue after the injection of a bolus of contrast agent. All inserts follow the same law, except for the amplitude. *C*_*i*_ (*s* ^−2^) determines the amplitude and was chosen in such a way that the maximum values of the enhancement were 10,18,26,34,42, and 50 HU. 

We first simulated acquisition and reconstruction with the reference protocol, consisting in full-scan T-FDK reconstructions [15]. *N*_sc_ = 40 scans were simulated with a rotation time of *T*_2*π*_ = 0.5 s during 40 s. The source was switched off every second rotation. Quantum noise was added to the projections. The corresponding values for the rotation time and smoothing parameter were calculated using (B.1) and (B.2) and are given in Table 1. The lowest sampling rate implies a slow rotation scanning, in which the signal is not oversampled and therefore *λ* = 0. In this case, TSA-TFDK reduced to the TIA-TFDK algorithm as described in [6]. The highest sampling rate implies a fast rotation scanning such that the signal is strongly oversampled and therefore a high value of *λ* is needed to adjust the cut-off frequency to *ν*_max_. The noise parameter for each simulation was adjusted according to the number of scans in such a way that the accumulated total dose was kept constant.

**Figure 7:**
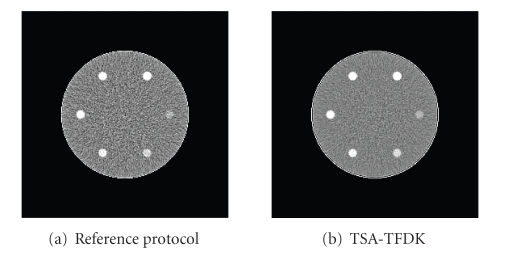
Comparison between reference protocol and low-noise reconstruction. (a) Frame of the sequence reconstructed with the reference protocol. (b) The same frame of the sequence reconstructed with fast scanning with TSA-TFDK. Window [30,70] HU.

**Figure 8:**
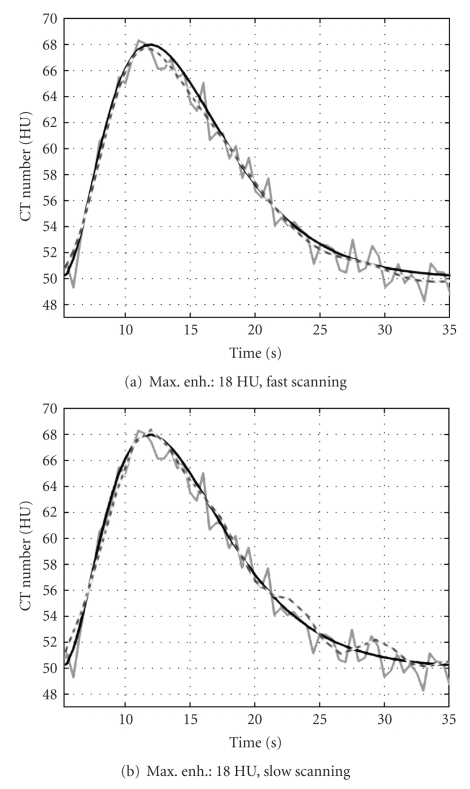
Time-attenuation curves of the inserts with maximum enhancement 18 HU. (a) Fast-scanning, (b) slow-scanning. Black curves: phantom value, grey curves: values obtained with the reference protocol, and dashed curves show the value of the reconstruction with TSA-TFDK.

Figures 7(a) and 7(b) show a frame of the reconstructed sequence with the reference protocol (left) and with TSA-TFDK with fast scanning (right). The noise reduction effect can be clearly observed. In Figure 8, we show the temporal evolution of the mean of the reconstructed attenuation values within the insert with maximum enhancement 18 HU. The curves of the TSA-TFDK sequences are clearly smoother than the curves obtained with the reference protocol, but the temporal resolution is preserved. The results with fast and slow scanning are qualitatively similar, except for some oscillations towards the end of the sequence in the slow scanned sequence, probably as a consequence of a light aliasing. 

 In Table 2, we show the value of the standard deviation measured within the inserts. As expected, the standard deviation in the TSA-TFDK reconstructions is lower than in the reference protocol sequence. The TSA-TFDK reconstructions of slow and fast scans exhibit a similar noise level. Indeed, according to (A.5) and (17) the ratio of the variances should be

(23) $$\begin{array}{c} r_{\sigma^{2}}\approx \frac{1}{2.3\nu_{\max\;}}=2.898. \end{array}$$

With the values from Table 2 we can calculate the measured reduction of the variance as 4.48/2.62 = 1.71 and (1.71)^2^ = 2.924, which shows that TSA-TFDK behaves as expected.

## 6. Results on Clinical Data

In this section, we show the effect of the low-noise reconstruction on a clinical data set. For this purpose, we present an example where the temporal smoothing is performed on the reconstructed images of a neurological clinical dataset. While this could be seen as a particular case of the TSA-TFDK algorithm for *N* = 1, we would like to remark that the TSA-TFDK has been developed for slow rotating scanners. This example is presented to illustrate the effect of the low-noise reconstruction and compare to current methods.

### 6.1. Data and Method

In the perfusion protocol data were acquired for 40 s, during which the source rotated at 0.5 s/rot and was switched on every two rotations. Hence, 40 images were generated which corresponds to a rate of 1 image/s. The scan was performed with a tube voltage of 120 kVp and at a tube current of 220 mA. Since the rotation time was *T*_2*π*_ = 0.5 s, the dose per reconstructed image in mAs was *D*_2*π*_ = 110 mAs. Every image frame was reconstructed from projection data of a full-scan with the reconstruction algorithm provided by the commercial scanner. At this point, we would like to point out that this study does not depend on the reconstruction algorithm, since data are processed after reconstruction. The X-ray beam was collimated to obtain a slice width of 10 mm. 

 Since we had already a reconstructed sequence at our disposal, we used it to estimate a value of *ν*_max_ instead of using an a priori general estimation for it. Note that this was possible because the sampling interval was *T*_*s*_ = 1 s which satisfies the sampling condition since *ν*_max_ ≪ 0.5 Hz [9]. For the purpose of estimating *ν*_max_, we used an ROI within the *arteria cerebri anterior*. The temporal evolution of the concentration of contrast agent in this artery can be assumed to be the fastest in the dataset. Additionally, the *arteria cerebri anterior* is approximately orthogonal to the slice plane so that partial volume effects are avoided. With this TAC we estimated *ν*_max_ as the value for which [−*ν*_max_, *ν*_max_] contains 99% of the energy of the signal. We obtained a value of *ν*_max_ ≈ 0.0966 Hz. 

 According to this estimation, the signal is clearly oversampled and there is leeway for low-pass filtering to increase the SNR as discussed in Section 3.2. Particularly, using (14) we get

(24) $$\nu_{c}^{\prime }=\frac{0.0966\;\text{Hz}\times T_{s}}{0.8}\approx 0.12$$

for which *λ* = 15.82 (see (13)). 

 As we saw in Section 3.1, this low-noise level can also be achieved by concentrating the same total dose to fewer rotations. A simple way to simulate sequences with different sampling rates is to downsample the original sequence by a factor of *K*, that is, to keep one every *K* samples of the original sequence. The maximum value of *K* can be obtained from (14) taking into account that *ν*_*c*_′ ≤ 0.5,

(25) $$\nu_{c}^{\prime }=\frac{\nu_{\max\;}KT_{s}}{0.8}\leq 0.5\to K\leq 4.14.$$

Hence, according to the analysis of Section 3.1, we should be able to obtain complete sequences with the same low level of noise from sequences sampled every 1, 2, 3, or 4 seconds. Using (13) and (25) we get *ν*_*c*_′ and *λ* for every sequence, these values are shown in Table 3. 

 Note that since we do not increase the dose by the same downsampling factor, the total dose applied *D*_tot_ is reduced by the downsampling factor *K*. The only way to avoid this would be to perform the acquisition of every sequence independently so that the dose per rotation is increased by the corresponding value for every sequence. Since this would be highly invasive for the patient, we use here a more simple way to verify the validity of our approach. We simply exploit the fact that the variance of the noise is inversely proportional to the dose applied as shown in (8). Thus according to our model, if we take as a reference the sequence with *K* = 1, the variance of the other sequences will be

(26) $$\begin{array}{c} \sigma_{K}^{2}=K\sigma_{\text{Spl}}^{2}\approx 2.3\bar{\varepsilon^{2}}KT_{s}\nu_{\max\;}. \end{array}$$

The variance of the noise of the original data is $\bar{\sigma_{Or}^{2}}=\varepsilon^{2}$. Hence, we can express the ratio between the variance of the noise in the original sequence and that of the downsampled and filtered sequences as

(27) $$\begin{array}{c} \frac{\sigma_{Or}^{2}}{\sigma_{K}^{2}}\approx \frac{1}{2.3KT_{s}\nu_{\max\;}}. \end{array}$$

### 6.2. Results

**Figure 9:**
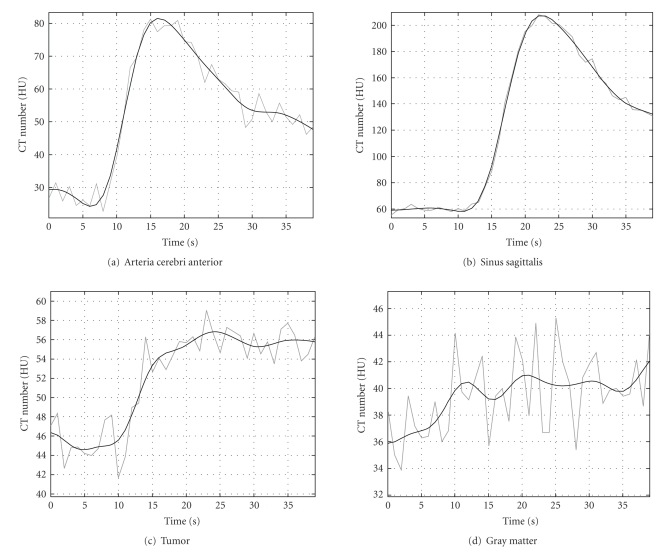
Example of TACs in different tissues. The gray curve corresponds to the original dataset. The black curve corresponds to the low-noise sequence.

In Figure 9, we show some examples of time-attenuation curves of the original (gray) and the low-noise sequence 1 with *K* = 1 (black). The curves show the temporal evolution of the average value of the pixels within a ROI in different tissues. As can be seen, the enhancement curve for the *arteria cerebri anterior* in the low-noise sequence is smooth but can still follow the changes in the original curve. The same can be observed for the *sinus sagittalis*. In such large vessels, the enhancement due to contrast agent flow is higher than in tissue or in small vessels, and the original SNR is high enough to compute physiological parameters without smoothing. In other regions, enhancement is larger than noise but of the same order of magnitude. An example for such regions is shown in curve Figure 9(c)) where the TAC of an ROI within a tumor is shown. The TAC of the low-noise estimation clearly eliminates noise and delivers a curve which is physiologically more plausible than the curve obtained from the original dataset. Finally, Figure 9(d)) shows the TAC of an ROI within gray matter. 

While in the original curve the enhancement cannot really be perceived, the TAC of the low-noise sequence clearly shows an increase with time in the concentration of contrast agent. On the other hand, the TAC of the low-noise sequence contains low frequency oscillations which are not of physiological nature. These are due to low-frequency noise in the frequency band [−*ν*_max_, *ν*_max_]. 

**Figure 10:**
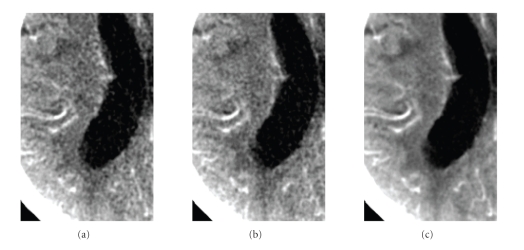
Detail of the left hemisphere in a frame of the perfusion sequence with high contrast enhancement (*t* = 19 s). (a) Original sequence. (b) Sequence 4 (*T*_*s*_ = 4 s). (c) Sequence 1 (*T*_*s*_ = 1 s). Window [16,56] HU.

**Figure 11:**
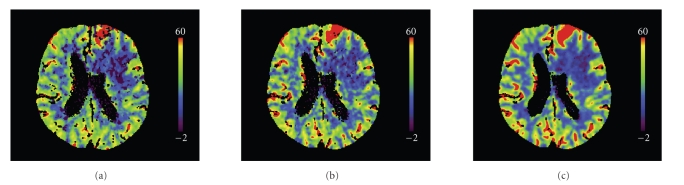
Cerebral blood flow maps computed from the original sequence (a), sequence 4 (*T*_*s*_ = 4 s), (b) and sequence 1 (*T*_*s*_ = 1 s) (c).

 We would like now to verify if the reduction of the noise caused by the temporal smoothing is in accordance with the predictions of our model. Noise is measured as the variance of pixel values in a certain region of a homogeneous object. The variance might be due not only to quantum noise but also to pixels in the region of interest that correspond to different tissues and have therefore a different temporal behavior. Since our model concerns the temporal behavior of the statistical fluctuations caused by quantum noise only, we must segment a region within a tissue where all points have the same temporal behavior. This is not practicable in clinical images since we do not know a priori the exact temporal behavior in any region, except for in those regions where the attenuation is constant because contrast agent does not reach them. For this reason we used a segmented ROI within the ventricular system to estimate the variance. This delivered a set of 2300 pixels with the same temporal behavior. We then used the whole 2300 × 40 pixels to estimate the variance in each reconstructed sequence. The ratio of the variance of the original sequence to the variance of the postprocessed sequences is shown in the second column of Table 4. The third column shows the values estimated using (27). The estimated values match approximately the measured values. According to the proposed model for the temporal behavior of noise, the reduction of the variance should be inversely proportional to the sampling rate since the total dose is decreased by downsampling. This is approximately in accordance with the values in Table 4. The effect on image quality is shown in Figure 10. The frame shown corresponds to *t* = 19 s. Sequence 4 has samples at *t* = 16 s and *t* = 20 s so that the frame at *t* = 19 s has been computed at the filtering step. The image quality of the frame estimated from sequence 4 appears to be equivalent to the original frame although four times fewer input data were used for the computation of the sequence. Sequence 1, with strong temporal smoothing, presents substantially reduced noise while visually preserving spatial resolution. The reconstructed sequences were used as input for the Perfusion CT software (Siemens AG, Healthcare Sector, Forchheim, Germany) that computes the functional parameter maps. This software first segments vessels and bone, performs a strong spatial smoothing, and subsequently computes the functional parameters. The segmented regions are excluded from the functional maps and represented in black. Figure 11 shows cerebral blood flow maps computed from the original sequence (a), sequence 4 (b), and sequence 1 (c). Map (a) presents many small isolated segmented regions compared to map (c). These correspond to areas where the noise level led to a wrong classification as vessels. Most of them disappear in map (c). Finally, map (c) is smoother in space which is physiologically more plausible. The quality of maps (a) and (b) is equivalent although (b) was computed from a sequence reconstructed with four times fewer data, equivalent to four times less dose. 

## 7. Conclusion

In this paper we have presented the dynamic reconstruction algorithm TSA-TFDK, which is a further development of the previously published TIA-TFDK algorithm [6]. A temporal sequence of image frames is generated. In the presented modification a smoothing term is incorporated into the temporal estimation step reducing the noise level. The continuous low-pass filter covers only the relevant bandwidth of the dynamic process and higher frequencies are strongly attenuated. No relevant information is lost. Image properties are independent of the sampling rate and the rotation time of the scanner as long as Nyquist's sampling condition is fulfilled. The obtained level of noise only depends on the total dose applied. As an example the TSA-TFDK allows to reconstruct a dynamic perfusion process with a physiologic relevant maximum frequency of *ν*_max_ = 0.15 Hz from data acquired by scanning with any rotation time up to 5.33 s. In a simulation study similar temporal resolution and noise level are achieved by acquiring with slow and fast rotation time. We have illustrated the effect of the low-pass filtering using a clinical data set, and the results obtained were in accordance with the predictions obtained from a model for the temporal behavior of noise in dynamic X-ray imaging presented. The proposed approach opens the possibility to use other tomographic X-ray techniques such as small animal imaging CT scanners for perfusion studies. It might even be a starting point for the development of dynamic reconstruction algorithms for C-arm systems by adapting the estimation step to the irregular sampling scheme caused by the back and forth movement. However, the assumption of no motion limits the possible clinical applications to fixed patients.

## Figures and Tables

**Figure 12:**
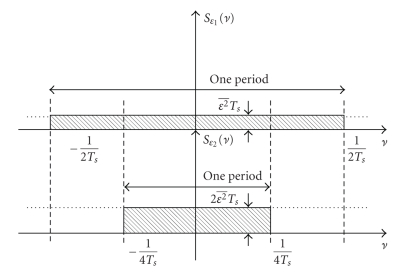
Physical power spectral density of uncorrelated sequences obtained by sampling a continuous stationary random process with sampling rates 1/*T*_*s*_ (top) and 1/(2*T*_*s*_) (bottom).

**Table 1:** Reconstruction parameters for slow and fast scanning reconstruction.

	Slow scanning	Fast scanning
*n*	9	9
*λ*	0	1533
*T*_2*π*_ (s)	5.33	0.5
*T*_*s*_ (s)	2.66	0.25
*N*	16	2
No. rotations	8	80

**Table 2:** Standard deviation of the values within the inserts for the reference protocol and the TSA approaches.

Algorithm	Std. dev.
Std. protocol	4.48
TSA-TFDK fast scanning	2.60
TSA-TFDK slow scanning	2.62

**Table 3:** Input sequence and filter parameters for every sequence.

Sequence	No. of images	*T*_*s*_(*s*)	*ν*_*c*_′	*λ*
Seq. 1	40	1	0.121	15.82
Seq. 2	20	2	0.241	0.0154
Seq. 3	13	3	0.362	2.57*e* − 4
Seq. 4	10	4	0.483	4.41*e* − 6

**Table 4:** Sequences and reduction of the variance.

Sequence	Measured	Estimated
1	4.90	4.5
2	2.32	2.25
3	1.54	1.5
4	1.04	1.12

## Appendices

### A. Temporal Sampling of Noise in Dynamic X-Ray Tomographic Imaging

We consider the temporal evolution of a single noisy projection value which we denote by *y*(*t*). This value is determined by the angular position of the source, the fan-angle and the cone-angle:

(A.1) $$\begin{array}{c} y\left(t\right)=P_{\alpha }\left(\gamma ,\varphi ,t\right). \end{array}$$

The temporal evolution *y*(*t*) is modeled as a continuous deterministic signal *p*(*t*) contaminated by additive noise *ε*(*t*)

(A.2) $$\begin{array}{c} y\left(t\right)=p\left(t\right)+\varepsilon \left(t\right). \end{array}$$

The noise is assumed to be a continuous random process with zero mean and mean square value $\bar{\varepsilon^{2}}$.

For the following analysis we consider quantum as the main source of noise in X-ray projections and neglect the contribution of electronic noise. This is a reasonable assumption for applications where the maximum width traversed by the X-ray beam is small as, for example, neurological applications. According to the Poisson law the mean number of quanta detected by a detector pixel fluctuates around a mean value $\overset{̅}{N}$ with a variance equal to the mean. As a consequence of the process of contrast agent flow, the mean number of detected quanta depends on time and therefore so does the noise variance. However, the increase in attenuation due to contrast agent flow is small compared to the overall attenuation of the passing X-ray beam. Hence, it is reasonable to assume that the noise variance does not depend on time (is constant) [9] and thus *ε*(*t*) is a wide-sense stationary process in time [17]. We use hereafter the term stationary process to refer to a wide-sense stationary process. Moreover, for a given projection value, the fluctuations around the mean at two time instants *t*_1_ and *t*_2_ are statistically independent.

In dynamic acquisition the projection value *y*(*t*) is measured once during every rotation. This can be seen as a temporal sampling of the signal *p*(*t*) contaminated by noise *ε*(*t*). Let us now analyze the sampling of stationary process *ε*(*t*). We denote by *ε*_1_[*n*] = *ε*(*n* *T*_*s*_) the discrete sequence of noise samples obtained by sampling *ε*(*t*) with a sampling rate of 1/*T*_*s*_. As mentioned above, the fluctuations at two different times are statistically independent and therefore also uncorrelated. This can be expressed by the discrete autocorrelation function *R*_*ε*_1__ as

(A.3) $$\begin{array}{c} R_{\varepsilon_{1}}\left[n\right]=E\left[\varepsilon_{1}\left[k\right]\varepsilon_{1}\left[k+n\right]\right]=\bar{\varepsilon^{2}}\delta \left[n\right], \end{array}$$

where *E* denotes the expectation value and *δ*[*n*] is the Kronecker symbol (*δ*[0] = 1 and *δ*[*n*] = 0 if *n* ≠ 0). The discrete power spectral density *S*_*ε*_1__′ is defined as (see [18])

(A.4) $$S_{\varepsilon_{1}}^{\prime }\left(e^{i2\pi \nu^{\prime }}\right)=\underset{n\in \mathbb{Z}}{\sum }R_{\varepsilon_{1}}\left[n\right]e^{-i2\pi \nu^{\prime }n}=\bar{\varepsilon^{2}}=\text{const}$$

where *ν*′ = *ν* *T*_*s*_ is the normalized frequency with *ν* as the physical frequency. The notation (*e* ^*i*2*π**ν*′^) indicates that it is the Fourier Transform of a discrete sequence and is therefore periodic (we follow the notational conventions of [18]). *S*_*ε*_1__′(*e* ^*i*2*π**ν*′^) owes its name to the fact that integrating it over one period of the normalized frequency, ] − 1/2,1/2[, yields the mean square value or variance of the noise. Downsampling *ε*_1_[*n*] by a factor of *K* (or sampling *ε*(*t*) with sampling interval *K* *T*_*s*_) would deliver a sequence with the same discrete autocorrelation as in (A.3) and therefore the same discrete power spectral density as in (A.4).

Definition (A.4), however, does not provide any insight into the physical frequency of the underlying continuous process. For this reason we define the discrete physical power spectral density *S*_*ε*_1__ of the samples of a continuous process sampled every *T*_*s*_ by introducing the change of variables *ν*′ = *ν* *T*_*s*_ in (A.4). Thus, we get

(A.5) $$\begin{array}{c} S_{\varepsilon_{1}}\left(e^{i2\pi \nu T_{s}}\right)=T_{s}\underset{n\in \mathbb{Z}}{\sum }R_{\varepsilon_{1}}\left[n\right]e^{-i2\pi \nu nT_{s}}=\bar{\varepsilon^{2}}T_{s}. \end{array}$$

Integrating (A.5) over one period on the physical frequency axis we obtain the mean square value or variance of the discrete process

(A.6) $$\begin{array}{c} \bar{\varepsilon_{1}^{2}}=\int_{-1/2T_{s}}^{1/2T_{s}}S_{\varepsilon_{1}}\left(e^{i2\pi \nu T_{s}}\right)d\nu =\bar{\varepsilon^{2}}. \end{array}$$

Therefore *S*_*ε*_1__(*e* ^*i*2*π**ν**T*_*s*_^) describes the distribution of power density over one period of the *physical* frequency axis. In order to simplify nomenclature, we will use hereafter the wording *power spectral density* to refer to the *physical power spectral density*.

As a consequence of (A.5) and (A.6), the discrete power spectral density of the discrete sequence *ε*_*K*_[*n*], obtained by downsampling *ε*_1_[*n*] by a factor of *K* (or sampling *ε*(*t*) with sampling interval *K* *T*_*s*_), is increased by a factor of *K* with respect to the discrete power spectral density of *ε*_1_[*n*] (see Figure 12)

(A.7) $$\begin{array}{c} S_{\varepsilon_{K}}\left(e^{i2\pi \nu KT_{s}}\right)=K\bar{\varepsilon^{2}}T_{s}. \end{array}$$

### B. TSA-TFDK

The TSA-TFDK is summarized in the following steps.

Algorithm TSA-TFDK (1) Choose rotation time such that

(B.1) $$\begin{array}{c} T_{2\pi }\leq \frac{0.8}{\nu_{\max\;}}. \end{array}$$

(2) Calculate *λ* for *n* = 9

(B.2) $$\begin{array}{c} \lambda ={\left(\frac{\pi \nu_{\max\;}T_{2\pi }}{0.8}\right)}^{-10}-\pi^{-10}. \end{array}$$

(3) Rebin cone-beam projections to fan-parallel beam

(B.3) $$\begin{array}{c} P_{\alpha }\left(\gamma ,\phi ,t_{\alpha }\right)\to P_{\beta }^{b}\left(u,v,t_{\beta }\right). \end{array}$$

(4) Divide every rotation in *N* sets of projections and reconstruct *N* partial block backprojections with the T-FDK algorithm

(B.4) $$\begin{array}{cc} & Q_{\beta }\left(u,v,t_{\beta }+kT_{2\pi }\right)\\ & \;\;=\int_{-u_{\max\;}}^{u_{\max\;}}P_{\beta }^{b}\left(s,v,t_{\beta }+kT_{2\pi }\right)w\left(s,v\right)g\left(u-s\right)ds,\\ & \zeta_{j}\left(x,t_{\left(\pi /N\right)(2j+1)}+kT_{2\pi }\right)\\ & \;\;=\frac{1}{2}\int_{(2\pi /N)j}^{\left(2\pi /N\right)(j+1)}Q_{\beta }\left(u^{\prime }\left(x,\beta \right),v^{\prime }\left(x,\beta \right),t_{\beta }+kT_{2\pi }\right)d\beta , \end{array}$$

where for all *j* = 0,…, *N* − 1, for all *k* ∈ *ℤ*. (5) Combine the *j*th and the (*j* + *N*/2)th sequences of partial block backprojections to a unique sequence

(B.5) $$\begin{array}{cc} & \zeta_{j^{\prime }}^{\prime }\left(x,t_{\left(\pi /N)(2j^{\prime }+1\right)}+kT_{\pi }\right)\\ & \;\;=\{\begin{array}{cc} \zeta_{j^{\prime }}\left(x,t_{\left(\pi /N)(2j^{\prime }+1\right)}+kT_{2\pi }\right), & k\text{even},\\ \zeta_{j^{\prime }+(N/2)}\left(x,t_{\left(\pi /N)(2j^{\prime }+1\right)}+kT_{2\pi }-T_{\pi }\right), & k\text{odd}, \end{array} \end{array}$$

where for all *j*′ = 0,…, *N*/2 − 1, for all *k* ∈ *ℤ*. (6) Estimate the values of the partial reconstructions at the output frame times with polynomial spline filtering

(B.6) $$\begin{array}{cc} {\underline{\zeta }}_{j^{\prime }}\left(x,t\right) & =\underset{k\in \mathbb{Z}}{\sum }\zeta_{j^{\prime }}^{\prime }\left(x,t_{\left(\pi /N\right)(2j^{\prime }+1)}+kT_{\pi }\right)\\ & \;\times \eta_{\lambda }^{n}\left(\frac{t-t_{\left(\pi /N)(2j^{\prime }+1\right)}-kT_{\pi }}{T_{\pi }}\right), \end{array}$$

where for all *j*′ = 0,…, *N*/2 − 1. (7) Accumulate the estimated *N*/2 partial block backprojections to obtain the time dependent attenuation map

(B.7) $$\begin{array}{c} {\underline{\mu }}^{r}\left(x,t\right)=2\overset{N/2-1}{\underset{j^{\prime }=0}{\sum }}{\underline{\zeta }}_{j^{\prime }}\left(x,t\right). \end{array}$$
